# Effect of behavioral inhibition system and childhood emotional neglect on serotonergic activity, negative affect, and rejection sensitivity in non-clinical adults

**DOI:** 10.1371/journal.pone.0207746

**Published:** 2018-11-20

**Authors:** Min Jin Jin, Wookyoung Jung, Myoung Ho Hyun, Seung-Hwan Lee

**Affiliations:** 1 Clinical Emotion and Cognition Research Laboratory, Inje University, Goyang, Republic of Korea; 2 Department of Psychology, Chung-Ang University, Seoul, Republic of Korea; 3 Department of Psychology, Keimyung University, Daegu, Republic of Korea; 4 Department of Psychiatry, Inje University, Ilsan-Paik Hospital, Goyang, Republic of Korea; Radboud University Medical Centre, NETHERLANDS

## Abstract

**Introduction:**

Behavioral inhibition system (BIS) has a strong genetic basis, and emotional neglect (EN) in childhood is one of many environmental experiences that can affect individuals. This study aimed to examine the effects and interaction between BIS and EN on central serotonergic activity and other negative affect and cognition.

**Methods:**

A total of 153 non-clinical volunteers (54 men and 99 women; average age, 27.72 years, standard deviation = 6.40) were included in the analyses. The Behavioral Inhibition System scale, Childhood Trauma Questionnaire, and negative affect and cognition (Beck Depression Inventory, State-Trait Anxiety Inventory, and Rejection Sensitivity Questionnaire) were measured. As a biomarker of central serotonergic activity, the loudness dependence of auditory evoked potentials was measured.

**Results:**

High EN was associated with higher loudness dependence of auditory evoked potential (LDAEP) levels and low EN was associated with lower LDAEP levels in high BIS people only. People with high EN people showed significantly higher levels of depression and state anxiety than did those with low EN. Moreover, of people with low BIS, those who had more EN experience had higher levels of rejection sensitivity than did those with less EN experience, while people with high BIS did not show different patterns of rejection sensitivity regardless of the difference of EN.

**Conclusions:**

This study revealed different effects on physiological (loudness dependence of auditory evoked potentials), intrapersonal (depression and state anxiety), and interpersonal aspects (rejection sensitivity) based on the interaction of BIS and EN. Our results suggest that the physiological and interpersonal aspects, but not the intrapersonal aspect, are significantly influenced by the interactive effect of BIS and EN.

## Introduction

Over the past decades, numerous studies have found that the interactive effect of genetic and environmental factors contributes to individual differences [1] in serotonin system markers [2], depression [2], anxiety [3], and sensitivity to rejection [4]. Although the DNA-sequence has been considered as an up-to-date method in recent studies of gene-environment interactions, there could be more practical ways to identify such interactions using non-molecular approaches [5]. This study is aimed to examine possible gene-environment interactions using sources that are more economical and easily accessible by many social scientists, including psychologists.

Gray proposed behavioral inhibition and activation systems [6] as innate dispositions which mediate genetic influence on individual differences, such as temperament, behavior, and emotions [7]. The behavioral activation system (BAS) and behavioral inhibition system (BIS), defined by Gray [8], are particularly essential functions of communication and human emotion [6]. There is substantial evidence of neurobiological mechanisms for each of these systems [9]. Notably, BIS inhibits behavior in response to signals of punishment, non-reward, and novelty [9, 10]. BIS is known to be heritable [11, 12] and to have basis of neurobiological and biological mechanisms [13, 14]. Therefore, BIS could be utilized as an expedient psychological assessment that strongly reflects genetic differences among individuals.

A factor that could be conceived as an environmental component that influence individuals is the experience of emotional neglect (EN) in childhood. EN is defined as emotional deprivation or the absence of a nurturing emotional environment in childhood [15]. EN is a particularly important environmental factor compared with other childhood adversities since it causes various severe emotional problems [16], brain dysfunction [17], and psychological and physical distress even after adjustment for childhood physical and sexual abuse [15]. Therefore, many studies have examined EN independently [18, 19]. Individuals with a history of EN during childhood have an attentional bias toward threatening stimuli [20] and experience emotional and physical distress, in both youth [21] and adulthood [15]. Although assessing EN of the Childhood Trauma Questionnaire (CTQ) relies on retrospective and subjective reports, the CTQ is well-validated and known to be stable overtime [22], and various studies used EN of CTQ as a measurement of environmental stressor [23–25]. Thus, EN could be employed as an element that is influenced by environmental experiences.

Both BIS and EN are related to serotonin activity. Behavioral inhibition is regulated by central serotonin activity [26]. BIS, which is sensitive to cues of threat, functions through serotonergic activity in the septohippocampal system [27]. The main role of serotonin in motivation is the inhibition of behavior [28]. A childhood history of EN can also influence the development of various neurobiological systems and can cause reduced serotonin activity in adulthood [29–31]. EN in childhood was reported to be associated with lower cerebrospinal fluid levels of the serotonin metabolite 5-hydroxyindoleacetic acid in adulthood [32].

The loudness dependence of auditory evoked potential (LDAEP), also known as the intensity dependence of auditory evoked potentials (IAEP), is an electroencephalography (EEG) measure and known to be inversely related to central serotonergic neurotransmission [33–36]. As a biomarker of serotonin activity, LDAEP may be associated with not only BIS but also EN.

In association with serotonin activity, BIS and EN could also be related to factors of intrapersonal distress such as depression and anxiety. Individuals with high BIS reactivity are expected to have higher levels of anxiety [27, 37, 38] and depression [27, 39, 40]. EN is also a predictor of emotional distress, including anxiety and depression in youth [21] and adults [15].

Both BIS and EN may be related to interpersonal factors such as rejection sensitivity (RS). RS is a cognitive-affective processing disposition in which the individual anxiously expects rejection [41]. From a social perspective, rejection includes aspects of punishment and increases the avoidance motivation that is salient for high BIS individuals [42]. In addition to BIS, a history of abuse may markedly alter an individual’s sensitivity to social rejection [43, 44].

While both BIS and EN could be related with various characteristics, it remains unclear how they are related or whether there is an effect of their interaction. Therefore, this study was designed to discover the main and the interactive effects of BIS and EN–constructs that can be assessed conveniently yet proposed to reflect genetic and environment influences, respectively–on individual differences. Measures of individual differences included LDAEP (physiological aspect), anxiety and depression (intrapersonal aspect), and RS (interpersonal aspect).

## Materials and methods

### Participants

A total of 157 healthy and non-smoking Korean volunteers recruited through advertisements in local newspapers were included in this study. Four subjects were eliminated because of missing data; thus, the final sample included 153 subjects. The population consisted of 54 (35.3%) men and 99 (64.7%) women with a mean age of 27.72 years (standard deviation [SD] = 6.40). The subjects had a mean of 14.44 years of education (SD = 1.78). Each participant provided a written informed consent, and the study was approved by the Institutional Review Board at Inje University Ilsan Paik Hospital before initiation (IRB no. 2015-07-026-001).

### Psychological measures

The Behavioral Inhibition System (BIS) scale from the Behavioral Inhibition System and Behavioral Activation System Scales, designed in accordance with Gray’s theory [45] and well-validated in Korea [46], was used. The BIS scale is a self-reported questionnaire consist of 7 items and is assessed using 4-point Likert scale ranging from 1 (“I strongly agree”) to 4 (“I strongly disagree”).

The EN scale was used from the Childhood Trauma Questionnaire (CTQ) which was validated in Korea [47]. The CTQ is a self-reported questionnaire that is reported to be well-validated and stable over the time [22]. The CTQ is used to assess childhood trauma, including emotional abuse, physical abuse, sexual abuse, EN, and physical neglect [48]. The EN subscale consists of 8 items and is assessed using a 5-point Likert scale that ranges from 1 (“never true”) to 5 (“very often true”). The score range of 5–9 is considered as none or minimal, 10–14 as low, 15–17 as moderate, and 18–25 as severe emotional neglect [49–51].

For the intrapersonal aspects, depression and state anxiety were measured. Depression was evaluated using the Beck Depression Inventory (BDI), developed by Beck [52] and validated in Korea [53]. It is composed of 21-items and assessed using a 4-point Likert scale ranging from 0 (‘‘almost never”) to 3 (‘‘almost always”). State anxiety was measured using the Korean-validated version of the State-Trait Anxiety Inventory [54], which was used to evaluate both state and trait anxiety [55]. It is composed of 40 items and assessed using a 4-point Likert scale ranging from 1 (‘‘almost never”) to 4 (‘‘almost always”). The subscale of state anxiety with only 20 items was used in this study.

RS was measured as an interpersonal aspect. Rejection Sensitivity Questionnaire (RSQ) was used to measure RS [56]. The RSQ consists of 18 hypothetical interpersonal situations with possible rejection by a significant other. Participants rated (1) the level of anxiety and concern about the outcome and (2) the perceived likelihood of acceptance or rejection by their significant other in each situation with 6-point Likert scale. The scores were calculated by first weighting the expected likelihood of rejection for each situation by the degree of anxiety, and then averaging these weighted scores across the 18 situations.

### Loudness dependence of auditory evoked potentials

Electroencephalogram data for all participants were acquired in a sound-attenuated EEG room using a NeuroScan SynAmps amplifier (Compumedics USA, Charlotte, NC, USA) with 64 Ag–AgCl electrodes mounted on a Neuroscan Quik-Cap (Compumedics, Charlotte, NC) using an extended 10–20 placement scheme. The ground electrode was located on the forehead, and the physically linked reference electrode was attached to both mastoids. The vertical electrooculogram (EOG) was positioned above and below the left eye, and the horizontal EOG was obtained at the outer canthus of each eye. The impedance was maintained below 5 kΩ. All data were processed with a 0.1–100 Hz band pass filter and sampled at 1000 Hz.

The recorded electroencephalogram data were preprocessed using CURRY 7 (Compumedics USA, Charlotte, NC, USA). Gross artifacts, such as those caused by movements, were rejected through visual inspection by a trained person who was blinded to the origin of the data. Artifacts related to eye movement or blinks were eliminated using the mathematical procedure implemented in the preprocessing software [57]. The data were filtered using a 0.1–30Hz bandpass filter and epoched from 100ms pre-stimulus to 900ms post-stimulus. The epochs were subtracted from the average value of the pre-stimulus interval for baseline correction. If any remaining epochs contained significant physiological artifacts (i.e., amplitudes exceeding ±75 μV) in any of the 62 electrode sites, they were excluded from further analysis.

Auditory stimulation included 1000 stimuli with an inter-stimulus interval randomized between 500 and 900 ms. Tones of 1000 Hz that were 80 ms in duration (10 ms rise and 10 ms fall) were presented through MDR-D777 headphones (Sony, Tokyo, Japan) at five intensities: 60, 70, 80, 90, and 100 dB SPL. These stimuli were generated by E-Prime software (Psychology Software Tools, Pittsburgh, PA, USA). For each subject, the N1 peak (the most negative peak between 50 and 200 ms from the stimulus) and P2 peak (the most positive peak between 150 and 300 ms from the stimulus) were subsequently determined at the Cz electrode [58, 59] for the five intensities. Cz was chosen as it is the best single electrode position for assessing activity of the auditory cortices [60] and the reliabilities at Cz can reach the same level as previously reported by dipole-source-localization methods [61]. The peak-to-peak N1/P2 amplitudes were calculated for each of the 5 stimulus intensities, and the LDAEP was calculated as the slope of the linear regression.

### Statistical analysis

Normality was tested for each variable before statistical analysis. Skewness > 2.0 and kurtosis > 7.0 were considered to be substantial departure from normality [62]. All variables in our results were within the range of normal distribution. After analyzing averages and SDs of variables, EN and BIS scores were dummy coded into two groups (low and high). Regression analyses were conducted with sex as a covariate to examine the main and interaction effects of the low/high EN and low/high BIS. Regression analyses were performed instead of analysis of covariance in order to control for the possible effect of sex, which was found to be different between the groups [63]. The significant level was set at p < 0.05 (two-tailed). Statistical analyses were performed using SPSS 21 (SPSS, Inc., Chicago, IL, USA) and regression analyses to examine interaction effects were performed using Process designed for SPSS [64].

## Results

### Descriptive statistics

The subjects were divided into four groups: low EN and low BIS score, low EN and high BIS score, high EN and low BIS score, and high EN and high BIS score groups. The high and low EN scores were divided based on a score of 10, which is the cut-off score between none and low severity of EN [48–51]. The high and low BIS scores were divided based on a score of 21, which was the median score of the study population. Table 1 presents the comparisons of demographic and psychological characteristics between the four groups.

**Table 1:** Comparison of demographic, psychological, and behavioral characteristics in participants with low EN and low BIS scores, low EN and high BIS scores, high EN and low BIS scores, and high EN and high BIS scores.

	Low EN & Low BIS (N = 39)	Low EN & High BIS (N = 25)	High EN & Low BIS (N = 50)	High EN & High BIS (N = 39)
	*Mean* ± *SD* or N (%)			
Age (years)	27.33 ± 6.92	26.20 ± 6.01	27.94 ± 6.35	28.79 ± 6.17
Sex				
Male	17 (43.6)	8 (32.0)	17 (34.0)	12 (30.8)
Female	22 (56.4)	17 (68.0)	33 (66.0)	27 (69.2)
Education (years)	14.31 ± 1.79	13.92 ± 1.87	14.48 ± 1.69	14.87 ± 1.77
Emotional Neglect (EN)	10.67 ± 2.11	10.44 ± 1.92	21.56 ± 5.12	21.31 ± 4.81
Behavioral Inhibition System (BIS)	19.46 ± 1.86	23.48 ± 2.04	20.26 ± 1.16	23.08 ± 1.13
LDAEP (μV/10dB)	1.0335 ± 0.543	0.808 ± 0.620	1.024 ± 0.557	1.252 ± 0.766
Beck Depression Inventory (BDI)	5.49 ± 4.09	6.76 ± 4.15	8.86 ± 6.20	9.79 ± 6.83
State Anxiety Inventory (SAI)	32.77 ± 8.06	35.28 ± 6.98	39.10 ± 7.57	38.00 ± 7.96
Rejection Sensitivity Questionnaire (RSQ)	8.32 ± 2.37	10.21 ± 2.96	10.31 ± 2.64	10.03 ± 2.81

EN, emotional neglect; BIS, behavioral inhibition system; LDAEP, loudness dependence of auditory-evoked potentials; BDI, Beck depression inventory; SAI, state anxiety inventory; RSQ, rejection sensitivity questionnaire; SD. Standard deviation

### Regression analysis

**Fig 1:**
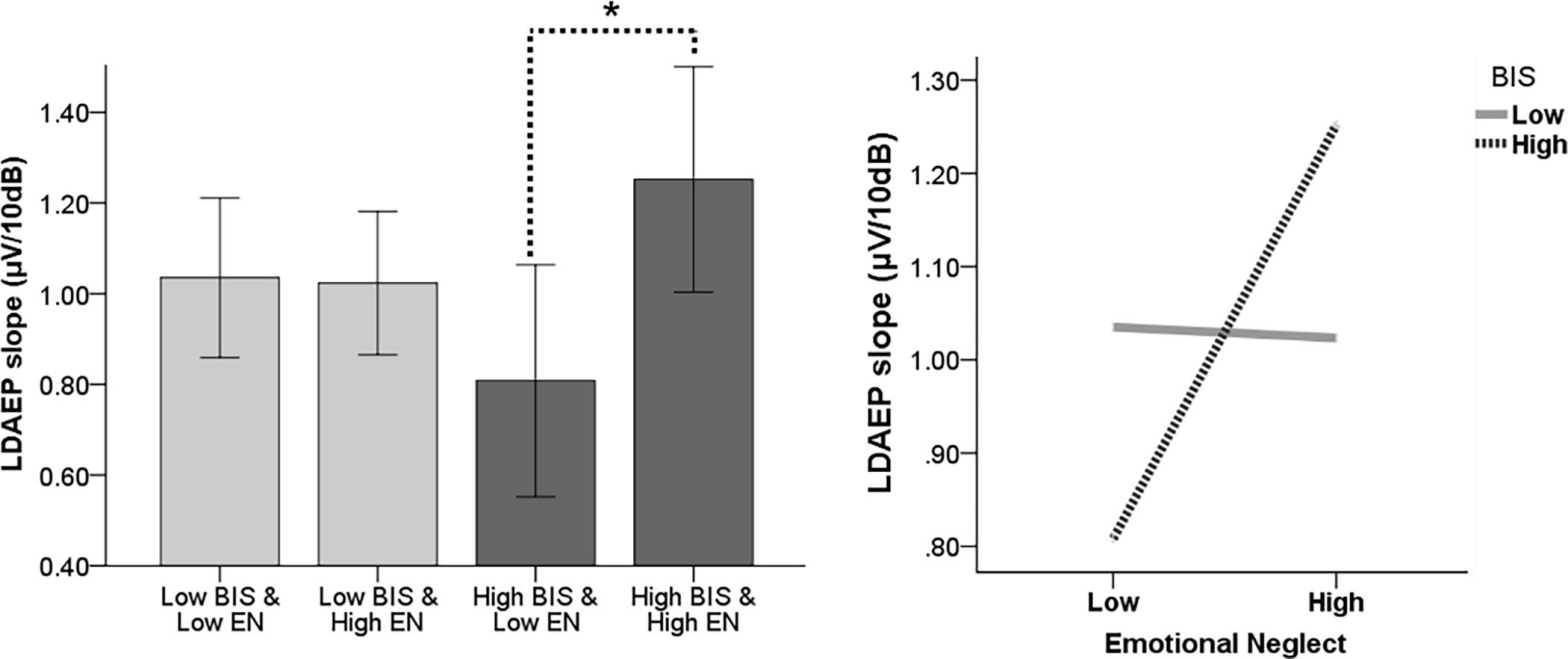
Interactive effect of the behavioral inhibition system and emotional neglect on the loudness dependence of auditory-evoked potentials.

In order to examine the interactive effect of the low/high EN and low/high BIS, regression analyses were performed with sex as a covariate. For the LDAEP slope level, the coefficients of EN (*B* = -0.51, *p* = .098) and BIS (*B* = -0.26, *p* = .109) were not significant. However, the coefficient of the interaction of EN and BIS (*B* = 0.48, *p* = .022) was significant (Table 2). The R^2^ was increased due to the interaction (*Δ R*^*2*^ = 0.03, *Δ F* = 5.37, *p* = .022). For subjects with low BIS score, the LDAEP level was not significantly different whether EN was low or high [*t*(87) = .10, *p* = .921]. However, for the subject with high BIS score, LDAEP level was significantly higher in those with high EN than in those with low EN [*t*(62) = −2.43, *p* = .018] (Fig 1).

**Table 2:** Results of regression analyses of EN, BIS, and the interaction of EN and BIS on LDAEP and RS.

	*B*	*SE*	*t*
	DV: LDAEP		
(constant)	.912	.277	3.292**
EN	-.510	.307	-1.664
BIS	-.255	.158	-1.614
EN×BIS interaction	.476	.205	2.318*
sex (covariate)	.242	.105	2.310*
	DV: RSQ		
(constant)	5.932	1.207	4.915***
EN	4.100	1.336	3.067**
BIS	1.848	.688	2.687**
EN×BIS interaction	-2.141	.894	-2.395*
sex (covariate)	.345	.455	.758

* *p* < 0.01.

** *p* < 0.01.

*** *p* < 0.001.

EN, emotional neglect; BIS, behavioral inhibition system; DV, dependent variable; LDAEP, loudness dependence of auditory-evoked potentials; RSQ, rejection sensitivity questionnaire.

For depression, all coefficients, including EN (*B* = 3.46, *p* = .218), BIS (*B* = 1.11, *p* = .441), and the interaction between EN and BIS (*B* = -0.22, *p* = .906) were not significant. Separate simple linear regressions were conducted to examine the effect of EN and BIS independently. There was significant effect of EN (*B* = 3.19, *p* = .001, *R*^*2*^_*corr*_ = .081), while the effect of BIS alone was insignificant (*B* = 1.12, *p* = .242, *R*^*2*^_*corr*_ = .016). In addition, for state anxiety, the coefficient of EN (*B* = 9.73, *p* = .013) was significant, yet coefficients of BIS (*B* = 2.38, *p* = .233) and the interaction of EN and BIS (*B* = -3.51, *p* = .175) were not significant. Results of simple linear regressions also revealed that the effect of EN alone was significant (*B* = 4.79, *p* < .001, *R*^*2*^_*corr*_ = .083), while the effect of BIS alone was insignificant (*B* = 0.50, *p* = .704, *R*^*2*^_*corr*_ = -.003).

**Fig 2:**
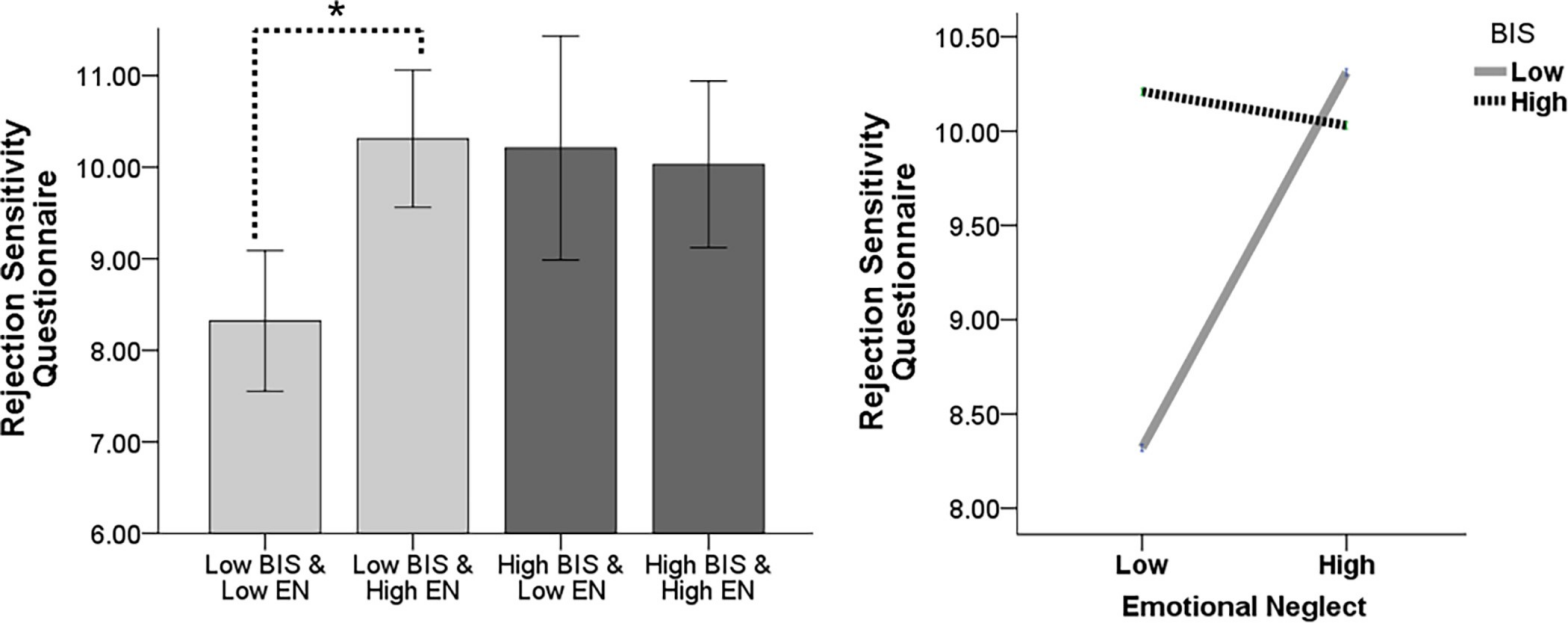
Interactive effect of the behavioral inhibition system and emotional neglect on results of the rejection sensitivity questionnaire.

For RS, all coefficients of EN (*B* = 4.10, *p* = .002), BIS (*B* = 1.85, *p* = .008), and the interaction of EN and BIS (*B* = -2.14, *p* = .018) were significant (Table 2). The R^2^ was increased due to the interaction (*Δ R*^*2*^ = 0.04, *Δ F* = 5.74, *p* = .018). The RS score was significantly higher in subjects with low BIS score and high EN than in those with low BIS score and low EN [*t*(62) = −3.69, *p* < .001]. However, there was no significant difference of the RS score depending on the level of EN in subjects with high BIS score [*t*(87) = .24, *p* = .808] (Fig 2).

## Discussion

This study aimed to demonstrate the effects of BIS and EN on physiological, interpersonal, and intrapersonal factors: the LDAEP, RS, and depression and state anxiety, respectively. While BIS was considered to reflect genetic influence substantially, EN was considered as one of the environmental experiences that can affect individuals. The results revealed that the interaction between BIS and EN influenced both LDAEP and RS. However, there was no significant interaction found for depression and state anxiety.

This study determined whether the LDAEP level was influenced by the interaction of BIS and EN. Because LDAEP is known to reflect central serotonergic neurotransmission inversely [34, 36], it is plausible that LDAEP was influenced by both BIS and EN. Central serotonin function is a key element of behavioral inhibition [26, 65], and the development of a functioning serotonergic system is negatively affected by exposure to early EN [17, 66]. Previous studies that examined the relationship between BIS/BAS and LDAEP discovered that LDAEP was positively correlated with BIS [67] and negatively correlated with BAS [68]. The present study tried to examine this relationship with regard to the history of EN, which reflects bad environment. The LDAEP level was low when the level of EN was low and it was high when the level of EN was high as well in people with high BIS (high susceptibility), whereas it was not different depending on the level of EN in people with low BIS (low susceptibility).

This diametric effect is also discovered with serotonin-transporter genes. A study of the serotonin-transporter-linked polymorphic region (5-HTTLPR) revealed that although individuals with two risk alleles proved most adversely affected, they showed the most positive outcomes when stressful life events were absent [69]. This diametric effect including the current result could be explained as the differential-susceptibility hypothesis, which is that the most vulnerable individuals who are affected by many kinds of stressors could be the very same ones those who reap the most benefit from environmental support, including the absence of adversity [70]. BIS, which is considered to reflect more innate temperamental features, activates responses of inhibition and avoidance [71] and controls sensitivity and responsiveness to threats via serotonergic activity [40]. Because people with a low BIS score (low susceptibility) show decreased behavioral inhibition and sensitivity to threats related to low reactivity of serotonin level regardless of EN, they could be less reactive to emotional adversities than people with high BIS scores and show comparable levels of LDAEP regardless of a childhood EN experience. In contrast, the subjects with high BIS scores (high susceptibility) showed increased behavioral inhibition and sensitivity to threats, related to high reactivity of serotonin, so that they could react with higher sensitivity to EN and even show less level of LDAEP when EN was not present.

Neither depression nor state anxiety was influenced by the interaction of EN and BIS. Simple linear regression analyses revealed that the levels of depression and state anxiety were influenced by EN alone. Subjects who experienced EN showed significantly higher levels of depression and state anxiety than did those who did not. Although the serotonin level is related to these emotions [72], emotional distress could be more affected by an individual’s personal history of emotional experiences [15]. Particularly, emotional adversities in childhood could result in insecure attachment, which can later influence depression and anxiety in adulthood [73, 74].

RS was observed to be predicted by EN, BIS, and the interactive effect of EN and the BIS. In the subjects with low BIS scores (low susceptibility) who had more EN experiences (bad environment) had higher levels of RS than did those with less EN experience. However, in the subjects with high BIS scores (high susceptibility), the experience of EN did not result in significant differences in RS. These results indicate that people with high BIS scores are inherently highly sensitive to rejection; therefore, the experience of EN does not result in any significant difference. In contrast, people with low BIS scores are highly sensitive to rejection, but only when they experience EN. This is dissimilar to intrapersonal emotional problems because RS is an index of interpersonal, rather than intrapersonal, issues. Indeed, distinct differences between the interpersonal and intrapersonal effects of emotion and motivation have been demonstrated [75, 76]. People with high BIS shows worry proneness [77], sensitivity to signals of punishments and threat [13, 40, 78], and avoidance behavior [10]. Because rejection is known to encompass the aspects of punishment and increase avoidance motivation [42], people with high BIS scores might react sensitively to rejection, irrespective of an EN experience. In contrast, people with low BIS scores generally are not sensitive to rejection unless they experience EN. Therefore, both BIS and EN play a critical role in RS.

This study has several limitations. First, although LDAEP is known to be a reliable indicator of serotonin, further studies comparing LDAEP with more direct measurement of central serotonin levels are necessary before drawing firm conclusions. Moreover, this study only used the old version of the BIS scale, since the newest version of the BIS/BAS questionnaire is not yet validated in Korea. In addition, the use of more genetic indices such as single-nucleotide polymorphisms and assessing objective environmental effect would be needed in further studies to ensure results of this study. Lastly, this study used RS as the only interpersonal aspect. There may be other interpersonal variables to examine the effects of the BIS and EN that should be considered in future studies.

In conclusion, this study is the first to investigate the interactive effect of the BIS and EN on LDAEP, anxiety and depression, and RS. LDAEP, which reflects the serotonin level, was influenced by the interaction of the BIS and EN such that high EN (bad environment) was associated with higher LDAEP, whereas low EN was associated with lower LDAEP in high BIS subjects (high susceptibility), but these relationships were not observed in low BIS subjects (low susceptibility). However, intrapersonal negative emotions, such as depression and anxiety, were more influenced in subjects with a history of EN. In contrast, RS was affected by the interaction of the BIS and EN, such that the experience of EN resulted in a higher level of RS, but only in subjects with low BIS scores. Our results reveal the different effects of BIS, EN, and their interaction on the various characteristics of individuals.

## Supporting information

S1 Table All data underlying the findings. LDAEP, loudness dependence of auditory-evoked potentials; SAI, state anxiety inventory; BDI, Beck depression inventory; CTQ, childhood trauma questionnaire; BIS, behavioral inhibition system; RSQ, rejection sensitivity questionnaire; EN, emotional neglect.
(XLSX)
